# Credibility of the Neutrophil-to-Lymphocyte Count Ratio in Severe Traumatic Brain Injury

**DOI:** 10.3390/life11121352

**Published:** 2021-12-07

**Authors:** Dorota Siwicka-Gieroba, Wojciech Dabrowski

**Affiliations:** Department of Anaesthesiology and Intensive Care, Medical University of Lublin, 20-059 Lublin, Poland; w.dabrowski5@yahoo.com

**Keywords:** TBI, brain injury, neutrophils, lymphocytes, NLR, mortality

## Abstract

Traumatic brain injury (TBI) is one of the leading causes of morbidity and mortality worldwide. The consequences of a TBI generate the activation and accumulation of inflammatory cells. The peak number of neutrophils entering into an injured brain is observed after 24 h; however, cells infiltrate within 5 min of closed brain injury. Neutrophils release toxic molecules including free radicals, proinflammatory cytokines, and proteases that advance secondary damage. Regulatory T cells impair T cell infiltration into the central nervous system and elevate reactive astrogliosis and interferon-γ gene expression, probably inducing the process of healing. Therefore, the neutrophil-to-lymphocyte ratio (NLR) may be a low-cost, objective, and available predictor of inflammation as well as a marker of secondary injury associated with neutrophil activation. Recent studies have documented that an NLR value on admission might be effective for predicting outcome and mortality in severe brain injury patients.

## 1. Introduction

**Figure 1:**
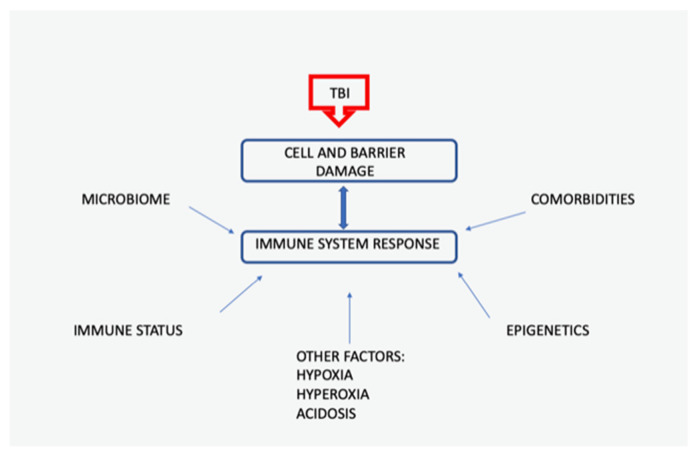
Selected factors that influence the immune response in TBI. The activity of the immune system depends on the homeostasis of the organism as well as on variable external factors; TBI-traumatic brain injury.

Traumatic brain injury (TBI) is one of the major causes of morbidity and mortality worldwide; it is a health problem that affects all ages and populations. Brain injury has two stages: primary injury due to mechanical damage and secondary injury that corresponds to a decrease in blood flow and oxygenation, edema, ischemic reperfusion injury, metabolic and endocrine dysfunction, oxidative stress, and impairment of ionic homeostasis. All these processes lead to activation and accumulation of inflammatory cells. The mechanism of inflammatory damage is still under investigation (Figure 1).

The immune cells, i.e., neutrophils, monocytes, as well as astrocytes and microglia, are crucial elements of acute cellular reactions. The inflammatory reaction after a TBI may be stimulated by damage associated with molecular patterns rapidly released after an injury. Disruption of the blood–brain barrier (BBB) is observed early after a TBI incident and a significant elevation in leukocyte migration aggravates a focal inflammatory response, leading to a worsening of secondary damage [1].

Several studies have reported that neutrophil count is usually elevated and lymphocytes present no significant changes during the acute phase after a brain injury [2,3]. These inflammation responses are closely correlated with poor outcomes for patients after injury.

The purpose of this article is to review the association between neutrophils and leukocytes and their neutrophil-to-lymphocyte ratio (NLR) related to outcome and mortality after a brain injury [4].

## 2. Neutrophils in TBI

Neutrophils, one of the crucial components of the innate immune system, mature from granulocyte/monocyte progenitor cells in the bone marrow. Neutrophils are infrequently observed in the central nervous system because of the BBB [5]. In a healthy person, a tight junction between endothelial cells stops neutrophils from penetrating the central nervous system [6]. A small number of neutrophils and other immune cells have been observed in cerebrospinal fluid (CSF), meninges, and pia membranes [6,7]. Under initial conditions, neutrophils exist only few hours. Although, human neutrophils in vivo may live more than 5 days [8]. Importantly, aged neutrophils present markers of a proinflammatory phenotype and other important changes as elevation of CXCR4 [9]. These processes are improving the neutrophils removal from circulation [10]

It is worth mentioning that neutrophils’ aging process is strongly controlled by microbiome [11]. Neutrophils are eliminated by macrophages in the spleen or liver, through apoptosis. Importantly, neutrophils activation can be changed by the surrounding environmental conditions, for example, hypoxia increases the lifespan of neutrophils [12].

Neutrophils have special defensive mechanisms for eliminating pathogens, which include matrix metalloproteinases (MMPs), myeloperoxidase, neutrophils elastase (NE), neutrophils gelatinase-associated lipocalin, and SGP28 [13,14]. Also, a neutrophil’s extracellular trap (NET) is another important protective mechanism [15]. Zhu et al. documented that NETs activation in the paraventricular nucleus is connected with sympathetic hyperactivity after traumatic brain damage. In animal model authors observed that reactive oxygen species in the paraventricular nucleus activate formation and chemotaxis of NETs which finally relate to the activation of microglia cells and increased secretion of IL-1β via the hippo/MST1 pathway [16].

In addition, neutrophils can intensify their own activation through an autocrine-dependent manner including leukotriene B4, IL-18 and platelet-activating factor [10]. Therefore, these cells are often phagocytosed or suppressed by macrophages or lymphocytes, because of the possibility of generating tissue injury [17]. Moreover, neutrophils release toxic molecules including reactive oxygen species (ROS), nitrous oxide (NOS), NADPH oxidase, proinflammatory cytokines, and proteases, which advance secondary damage after recruitment to an injured central nervous system (Table 1).

Under the pathological conditions of brain trauma, the number of neutrophils entering into the brain increases. Neutrophils are one of the earliest myeloid-derived cells that infiltrate the injured brain and affect secondary damage [36]. Neutrophils enroll to the injured site of the brain within 1 h of a focal injury, damage the parenchyma, and provoke secondary cellular injury and brain tissue damage [37,38]. 

The peak number of neutrophils that infiltrate the brain is observed 24 h after an injury, but cells infiltrate along the arterioles and venules within 5 min of closed brain damage [39,40]. The liver is the main center of the systemic response of neutrophils after brain damage. The production of host cytokines is a response to inflammation in a brain craving the maturation and granulation of neutrophils [41].The homeostatic feedback loop, the IL-23/IL-17/G-CSF axis that supervises neutrophil death and birth, is elevated after a brain injury [42].

Thus, what is the role of neutrophils in brain damage?

Free radicals induce disturbances of crucial transmembrane, tight-junction proteins such as occludin and claudin via the PI3K/AKT pathway, and finally, contribute to the break-up of BBB integrity and massive flux of molecules and cells across the barrier [43]. Neutrophil elastase is rapidly exhausted from activated neutrophils into the extracellular space, which intensifies the host inflammatory response. In addition, it can degrade the diversity of matrix and non-matrix proteins, such as proteins in plasma, proinflammatory mediators, or adhesion receptors [44,45]. These destructive effects of neutrophil elastase are observed though the early stage of a nervous system injury.

In addition, neutrophil-derived cytokines vary according to the underlying stimuli and tissues involved [46,47]. The specific variations of exterior phenotype, chemotaxis, and phagocytosis, which differ with classical inflammation, are observed according to a brain injury [48]. In neutrophils under brain injury status, in addition to normal proinflammatory cytokines, TNF, or chemokines CXCL-1, other anti-inflammatory cytokines or neutrotrophic factors can be found [49,50].

**Figure 2:**
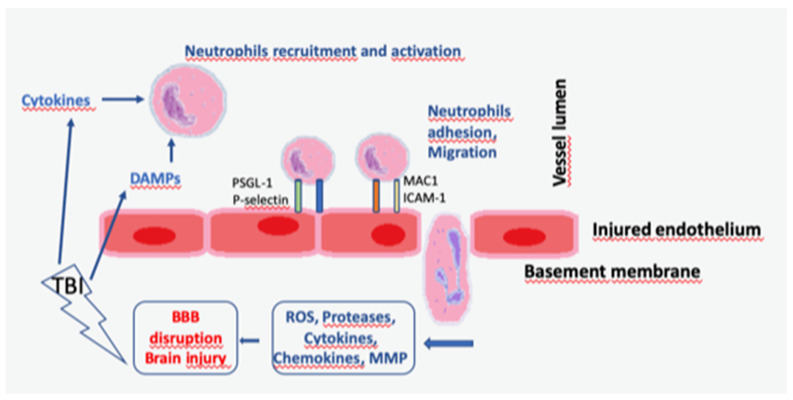
Neutrophil activation and circulation in TBI. After a TBI occurs, cells apoptosis and DAMPs leak into the extracellular spaces and activate immune cells. Activated neutrophils migrate towards the site of the injury. The elevated expression of adhesion molecules in endothelial cells, such as ICAM-1 or VCAM-1 1, increase the attachment and extravasation of peripheral immune cells into the central nervous system. Neutrophils (but also T cells and monocytes) penetrate the BBB. Neutrophils release toxic molecules including reactive oxygen species (ROS), nitrous oxide (NOS), NADPH oxidase, proinflammatory cytokines, and proteases, which advance secondary damage: DAMPs—danger/damage associated molecular patterns; ICAM—intercellular adhesion aolecule; MAC—membrane attack complex; MMP—matrix metalloproteinase; PSGL—P-selectin glycoprotein ligand; TBI—traumatic brain injury.

Cerebral hypoperfusion intensifies the interactions of neutrophils with blood vessels. Previous studies have indicated that hypoperfusion advances neutrophils to collapse and attach by elevating the expression of L-selectin and intercellular adhesion molecule 1 (ICAM-1) in vascular endothelial cells [51]. Activated neutrophils organize pseudopods and attach to the endothelium and other structures such as platelets, finally inhibiting blood flow through the microvascular system and elevating the risk of ischemia and early coagulopathy [52,53,54] (Figure 2).

In addition, vascular failure after neutrophils rolling is observed during and 4–8 h after injury [55]. Recent studies documented the important connection between neutrophils and hemorrhagic areas. Elevated activation of MMP-9 with basal lamina collagen IV mortification strongly amplifies the dysfunction of vessel integrity and development of hemorrhagic lesions [56]. Significantly, the aforementioned cells may induce iNOS 24–48 h after damage, which corresponds with hemorrhage and the dilution of vessels. One of the important questions is about the role of neutrophils in the conversion of hyperperfusion and hypoperfusion in the early stages after brain damage [57,58].

Another important aspect of neutrophil function after brain injury is infiltration of choroid plexus and CSF circulation of these cells, close to sites of damage. The BBB breakdown predisposes the stronger activation of neutrophil migration to the central nervous system [59]. The selectins act as rolling cells and connect with ICAM-1, which is expressed in peripherial vessels and platelet–endothelial adhesion molecules (PECAM-1) expressed in the chyroid plexus, and modulate the migration of neutrophils. The potential inhibition of ICAM-1 and PECAM-1 may improve neuroinflammation treatment. In addition, CXCR2 ligand expression increases the forcing of the aforementioned cells to parenchyma [60].

The above-mentioned formation of NETs intensifies neurological damage after a brain injury. A recent experimental study showed that NET formation was associated with hypoperfusion and tissue hypoxia and a decreased level of circulating NETs correlated with elevated serum deoxyribonuclease 1 (DNase-I) activity in brain injury. Furthermore, TLR4 and kinase peptidylarginine deiminase 4 (PAD4) moderates NET formation and cerebral microvascular dysregulation [15,48].

Neutrophils also damage the tight junction and permeability of BBB. The neutrophil-released NE breakdown the cadherin–cadherin binding and are finally predisposed to BBB hyperpermeability as well as neutrophil-derived MMPs, such as MMP2, MMP3, MMP9, which dysregulate the blood–brain barrier after central nervous system injury [61,62].

As mentioned above, BBB breakdown connects neutrophils with the pathophysiology of edema. Recent data showed that inhibition of Na^+^/H^+^ pump potentially decreases the cerebral infarct volume and neutrophil accumulation [63].

In summary, neutrophils are mediators during the early phase of secondary damage, exacerbate BBB damage, and promote the death of neuronal cells. Importantly, neutrophils are also determinants of long-term neurological recovery. A recent experimental study showed that the anti-CD11d treatment decreased macrophage and neutrophil activation and significantly improved outcomes focused on cognition, sensorimotor ability, and anxiety [64].

Mukherjee et al., in a large prospective study of isolated pediatric TBI patients, determined that a white cell count >16.1 × 109/L and a neutrophil count >11.9 × 109/L have predictive value for hospital length of stay and poor Pediatric Cerebral Performance Category Scale scores [65]. It is also worthwhile mentioning that a recent study by Dolmans et al., that demonstrated routine blood tests such as WBC and neutrophil count measured after severe TBI, upon admission, were not predictive of 30-day mortality, hospital length of stay, or outcome after 3 months [66].

## 3. Lymphocytes in Brain Injury

Peripheral immune system suppression is a predisposition for lymphopenia, which takes place in almost 80% of TBI patients [67]. Petrone et al., reported different lymphocyte dynamics in mild and severe TBI patients in the first 48 h after TBI. The elevation of lymphocyte count was significantly more dynamic in mild TBI especially after 48 h [68].

The effect and role of T lymphocytes in brain injured patients still remains to be investigated. A recent study showed that mainly adaptive immune response T cells infiltrate the site of a lesion. This process is increased by ROS released from neutrophils and occurs simultaneously with monocyte and macrophages [69].

Experimental studies on TBI and blood and brain tissues have reported the proliferation of naïve T lymphocytes, the polarization of effector T lymphocytes, and finally, suppression of the production of regulatory cells [52]. Peripheral M1 macrophages migrate towards the central nervous system and activate T cell proliferation and evolution to TH1 and TH17 proinflammatory subtypes. The γδ T and Th17 cells activate a proinflammatory microglia via modulating the FasL/PTPN2/TNF-α pathway. These mechanisms aggravate ischemic brain injury [70]. A concomitant reduction in Foxp3 + TREG production and elevation of the TH17/TREG ratio for weeks after a TBI are indicators of long-term adaptive immune responses. In addition, some studies have documented that T lymphocytes may not represent an important place in the pathogenesis of primary destruction in the brain during the first seven days after an injury [51].

A murine model has shown that consumption of regulatory T cells impairs T cell infiltration into the central nervous system, elevates reactive astrogliosis, and interferon-γ gene expression [46]. In addition, these autoreactive T lymphocytes probably induce the process of healing in an injured brain [52,54].

Recent evidence suggests that lymphocytes present particular relevance for lesion development. The data from an animal study indicated that lymphocyte-deficient RAG1 mice were secured from cortex stab wound damage [71]. The T lymphocyte CD4+ inhibitory agents significantly decreased acute damage of an injured brain in mice. Lymphocyte CD4+ participates in apoptosis activation in brain cells by cytokine production as TNF alpha or by FAS dependent pathways [72]. Prolonged immune system activation in the brain generates neurodegeneration and, finally, causes neurological disturbances [53]. The prevention of T cell migration to the central nervous system probably decreases neurodegeneration [73]. The elevation of effector/memory CD8+ T cells, which precedes interleukin-17/CD4+ T cell infiltration, has been connected with escalating neurological and motor impairment, increased of circulating brain-specific autoantibodies, and myelin pathology. An inadequacy or decrease in CD8+ T cells improved outcomes [70].

In addition, the CD3+/CD4−/CD8− T cells (double-negative T cells), known as γδ T cells, regulate the immune system and inflammatory homeostasis, and the number of these cells is significantly increased in a time-dependent manner in the central nervous system and peripheral blood [74,75]. However, Th17 cells present a crucial role in the secondary injury in the chronic phase after ischemic stroke. Furthermore, these cells are observed 1 week after traumatic brain injury (TBI) and intensified the cytotoxicity of CD8+ T cells at a next stage; therefore, the CD8+ T cell detrimental effect was observed [70,76]. Recent studies have documented that a stimulated level of IL-17 in peripheral blood samples is associated with worse final neurological outcomes. An elevated level of IL-17 has been observed in the peripheral blood 3 days after stroke [77,78]. It remains to be explained which types of immune system cells are responsible and crucial for stimulation of IL-17 [79,80]. In addition, a reduction in peripheral lymphocyte activation inhibited neurodegeneration and a decrease in lesion size was observed after a TBI [81].

The above-mentioned studies, indicate the need for novel methods of treatment after trauma, including influencing the cells of the immune system. A recent study by Nowell et al., showed that antagonizing of macrophage migration inhibitory factor (MIF) presented anti-neuroinflammatory and neuroprotective effects [82]. The results showed that administration of the MIF antagonist, as early as 30 min after an injury, prevented astrogliosis and growth of γδ T cells in the gut, inhibited the percentage of B cells infiltrating the brain, and finally predisposed the patient to a decrease in post-traumatic syndromes such as epilepsy.

## 4. NLR in TBI

The NLR is a rapid and simple parameter of inflammation. Several studies have reported that neutrophil count is usually elevated and lymphocytes presented no significant changes during the acute phase after a brain injury [2,3].

In addition, neutrophils are one of the fastest responders and mediators of secondary pathophysiology processes after TBI [83]. Lymphocytes are also very dynamic immune cells. Mrakovcic-Sutic et al., documented that the percentage of cytotoxic lymphocytes significantly decreased at day 1 and 4, and their number elevated at day 7 [84].

In recent years there has been a growing interest in NLR as a barometer of inflammation and a marker of secondary injury associated with cell activation and immune system response.

**Figure 3:**
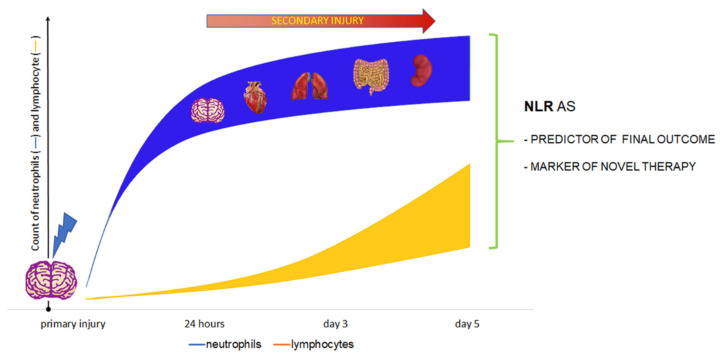
The place of the NLR in clinical management and final outcome prediction after acute brain injury. The peak number of neutrophils that infiltrate the brain is observed 24 h after an injury. The elevation of lymphocytes count is significantly more dynamic, especially after 48 h.

Brain trauma activates a cascade of various immune processes in the central nervous system. It is worth mentioning that the brain–multiorgan interactions seems to be important, because the brain is a central controller of the body. These interactions include relationships between brain and heart, lung, liver, kidneys and microbiome [85]. Brain damage increases the risk of complications from the above-mentioned organs and systems, increasing mortality in this group of patients. The immune system plays a critical role in this interaction as well as specific bidirectional immune system and brain axis [86]. Activation of neutrophils and lymphocytes in acute brain damage affects the imbalance in communication between the immune system and central nervous system [87] (Figure 3).

## 5. Prognostic Value of the NLR in TBI

Several studies have reported that the NLR may be a low-cost, objective, and available predictor of outcome in certain groups of patients, for example, in sepsis, lung cancer, pancreatic cancer, pancreatitis, hepatocellular carcinoma, pulmonary embolism, or cardiovascular diseases. Huang et al., reported that NLR is a helpful prognostic biomarker in sepsis and elevation of this value predisposes the patient to an unfavorable outcome in this group of patients [65,88]. In addition, NLR may also become a powerful presage of the inflammatory status and prognosis conditions of patients with glial tumor, ischemic, and hemorrhagic stroke and status epilepticus [89,90,91,92].

The outcome for patients after a TBI correlates significantly with age, pupillary reaction, Glasgow Coma Score (GCS) at admission, type of damage, coagulopathy as INR, and APTT. Recent studies have shown close correlations between final outcomes and total white blood cells (WBC), count lymphocyte ratio, count neutrophil ratio, NLR, platelet count, or platelet-to-lymphocyte ratio. Several studies have presented that a NLR value at admission might effectively predict outcome and mortality after a severe brain injury. The NLR potentially outperformed the predictive value of WBC, absolute neutrophil count (ANC), and absolute lymphocyte count, and is a reliable dynamic index of systemic inflammation that combines adaptive and innate immune system response pathways [59,74].

Importantly, the NLR may be a predictor of an hyper-acute inflammatory response as secondary damage and represents several advantages in the prediction of patients with severe brain injury.

### 5.1. NLR and Mortality

In a retrospective study, Chen et al., documented that the NLR amount on admission might be effective for predicting the one-year outcome and mortality in severe brain injury patients [93]. Similar conclusions by Huang et al. showed that a group of patients with a diagnosis of acute SAH, where increased red blood cell distribution width (RDW) and NLR were connected with higher observed one-year mortality, had an adjusted hazard ratio 1.03 (95% CI, 1.00–1.05) for per 1 increased NLR [94].

Significantly higher NLR in patients who died, shows that increased NLR, as well as age on admission, significantly predict the worse one-year outcome (age: OR = 1.068, 95% CI 1.052–1.083 and NLR: OR = 1.100, 95% CI 1.064–1.138, *p* < 0.9038) [68].

An additional study documented that an NLR value on admission higher than 15.63 has been shown to be an effective indicator of 28-day mortality and was greatly higher in DAI (diffuse axonal injury) patients as compared with CE (hemispheric or focal cerebral edema), ICH (intracerebral hemorrhage), and S-EH/SAH (epidural and/or subdural hematoma/subarachnoid hemorrhage) groups [95]. Another multivariate analysis revealed that NLR at admission was an independent prognostic factor of six-month outcome in a group after severe brain trauma (OR 0.91, 95% CI 0.89–0.93, *p* < 0.001) [96].

### 5.2. NLR and Clinical Outcome

Kimball et al. observed, in general, that higher NLR values 24 h and 48 h after TBI in pediatric patients were associated with worse final outcomes [97]. Recent results showed a higher NLR association with the Glasgow Outcome Score (GOS) or Glasgow Outcome Scale Extended (GOSE) [95,96,97]. Giede-Jeppe et al., described that NLR ≥7.05 is a good cut-off value to predict an unfavorable outcome, which indicates an mRS score of 3–6 after 3 months [98]. Further analyses performed by Wang et al., also found the positive correlation between the NLR and the Hunt–Hess grade. Authors showed that NLR may predict the adverse final outcome of patients with an mRS score of 3–5 after 3 months [99].

An elevated NLR is also an independent predictor for complications in patients with acute ischemic stroke (AIS), such as cerebral edema hemorrhagic transformation and ICH after endovascular treatment [100,101,102].

Additionally, its high value could predict functional independence, death, and risk of post-ischemic intracranial hemorrhage independent of age, treatment, and degree of recanalization [103,104].

The previously mentioned study by Mukherjee et al., also reported that an NLR above 5.2 predisposed a patient to a longer length of hospitalization and that Pediatric Cerebral Performance Category Scale (PCPCS) scores were less favorable. In addition, the NLR was after the GCS value as the next important independent risk marker for a worse outcome [65].

In recent years, one of the scientific questions has been, “Are NLR changes more objective than GCS changes?” Generally, the conditions reflected by the NLR and, importantly, the GCS assessment, are sedated, ventilated patients with severe periorbital swelling.

The power of the NLR for predicting the functional outcome of a brain injury may be very useful in relation to an outcome predicted by the Glasgow Coma Scale. Several studies have documented that higher NLR values are associated with lower GCS scores. Chen et al., summarized that the performance of the NLR for predicting a functional outcome in severe TBI was similar to GCS scores (AUC = 0.719, 95% CI 0.673–0.766). Nonetheless, some studies have highlighted that NLR is a weaker predictor of mortality in TBI patients than GCS [73,93,105].

Furthermore, other studies have noted that the impact of NLR on functional outcome should be analyzed in relation to pre-existing comorbidities versus whether it represents an independent causal relationship in the context of the observed immunosuppression [76]. In addition, Aexiou et al. investigated the role of NLR on the admission of brain-injured patients for predicting the need for CT scans in mild TBI. This study reported that higher NLR levels correspond with positive CT findings in mild TBI and suggested that a cut-off value of 2.5 could be used to quickly detect the need for a CT scan in mild brain injury with 78.1% sensitivity and 63% specificity [106].

### 5.3. NLR as Not Good Predictor of Mortality and Morbidity

However, in opposition to the above-mentioned studies, another recent study has documented that, in addition to the IMPACT and CRASH prognostic models, the NLR value at admission was not a good predictor of mortality and morbidity in moderate and severe TBI (AUC 0.58 and AUC 0.47) [107]. In addition, Corbett et al. reported that the NLR value was not significantly correlated with a risk of unfavorable outcome (AUROC, 0.500, *p* = 0.998) [108].

## 6. Neutrophils as a Target of Future Therapies

Neutrophils play an important role in many diseases such as pulmonary, cardiovascular autoimmune, infectious diseases, sepsis, and, the subject of this review, neuroinflammation and neurodegenerative diseases. Several studies are aiming to control neutrophils, their production, accumulation, or crucial changes in phenotype as a novel target therapy. For example, the CXCR2 antagonists’ effect is documented in a group of patients with asthma [109]. Other products, such as neutrophil elastase inhibitor, present positive properties in bronchiolitis obliterans [110]. The NETs effect is another promising future strategy. A crucial enzyme for intravascular and intraparenchymal neutrophil extracellular traps (NETs) formation—peptidylarginine deiminase 4 (PAD4)—is strongly activated in ischemic brain and overexpression predisposition to NET production and finally to decreased neovascularization and BBB disruption. Potentially, NET formation blockade by a PAD inhibitor may improve recovery [111].

Thus, therapeutic methods targeting neutrophils can involve many strategies such as increasing circulating neutrophil number (manipulation of the CXCL-12-CXCR-4 pathway), expressing their inhibitory receptors, targeting neutrophil production and activation by G-CSF, promoting neutrophil apoptosis, blocking chemokine functions, blocking neutrophils delivered mediators, or using selectin or integrin blockers [112,113,114,115,116,117]. As shown in Table 1 neutrophils may become a target in therapy after brain injury. Recent data showed that targeting DNA sensor cyclic GMP-AMP synthase (cGAS) or NETs potentially benefitted thrombolytic therapy in patients with ischemic stroke by decreasing tPA-associated hemorrhage [118].

## 7. NLR—A Potential Marker of Future Therapies in Chronic Neuroinflammation

Primary brain injuries impact crucial cellular processes causing secondary cell death mechanisms such as mitochondrial dysfunction, oxidative stress, BBB disruption, and chronic inflammatory processes. Chronic traumatic brain injury, similarly to acute TBI, includes neuroinflammation and, finally, contributes to long-term disabilities. In addition, in primary brain damage, neuroinflammation is helpful by stimulating an anti-inflammatory response. Sequentially, in the chronic brain injury stage an uncontrolled inflammation stimulates the pathological symptoms and follows the initial damage even 17 years after TBI [119]. Chronic neuroinflammation is connected with endogenous repair mechanisms and immune cells, microglia, cytokines, and chemokines. This inflammatory cells, neutrophils, monocytes, and lymphocytes cross the BBB and release prostaglandins and pro-inflammatory cytokines. Inflammatory regulators recruit microglia and immune cells to the brain by activating the expression of chemokines and cell adhesion molecules. Thus, NLR may be a useful, low cost marker, showing the peripheral inflammation and its correspondance with neurodegeneration [120,121,122,123]. In addition, chronic neuroinflammation may become a potential target for novel therapy as a select method of conversion from pro-inflammatory to anti-inflammatory reactions. Several studies focus on methods to intensify the protective effects of inflammation. Recent publications show positive effects of several drugs such as minocycline, melatonin, statins, subdural infusion of serpine-1, mesenchymal stem cells therapy [124,125,126,127,128,129]. All of these therapies can modulate a neuroinflammatory response, which may affect NLR.

Although several studies have described the credibility of NRL in the prognosis of the outcome and efficacy of revascularization in ischemic stroke patients, its usefulness in assessing treatment efficacy has been poorly recognized in patients with TBI. A lot of studies have documented its predicting value in response to treatment in cancer and immunomodulation therapies [130,131,132]. Gudson et al., showed a strong correlation between baseline NLR and 24-h growth of perihematomal edema in patients treated for intracranial hemorrhage, however they analyzed only a relationship between the expansion of cerebral edema and NLR [133]. Changes in NLR in TBI patients were analyzed by Petrone et al. [68]. Severe TBI caused an increase in NLR above 5.0 at 48 h of treatment, whereas its values decreased following treatment in patients treated for mild TBI. Based on their results it can be speculated that an increase in NLR results from a neuroinflammatory response to trauma and a non- or poor response to treatment, however they did not analyze the changes in NLR as the efficacy response to treatment. It is worth stressing that the analysis of changes in NLR as a response to treatment seems be very useful in TBI patients as it can modulate a treatment before critical events.

Regulation of the activation and deactivation of immune cells and the above-mentioned processes may be important in the brain’s recovery. Potential strategies in sequestering inflammation and its associated neurodegenerative processes need future study.

## 8. Conclusions

The NLR is a simple and low-cost index of systemic inflammation status and has been shown to have prognostic value for predicting poorer functional outcomes and elevated mortality rates in TBI patients. However, the NLR differentiation of TBI types needs further study.

## Figures and Tables

**Table 1:** Selected neutrophil-derived interleukins and chemokines, their functions in a brain injury and potential therapeutic methods of treatment. Neutrophils release toxic molecules including reactive oxygen species (ROS), nitric oxide synthase (NOS), NADPH oxidase, proinflammatory cytokines, and proteases, which advance secondary damage; BBB—blood brain barrier; CXCL—chemokine (C-X-C motif) ligand; IL—interleukin; JNK—c-Jun N-terminal kinase; NLRP—nucleotide-binding oligomerization domain; TGF—transforming growth factor.

Name	Effects	Therapeutic METHODS	References
Interleukins			
IL-1α	BBB breakdown; apoptosis angiogenesis	Recombinant human IL-1Ra, NLRP3 inhibitor, Mesenchymal stem/stromal cells therapy, ketamine	[18,19,20]
IL-1β	Apoptosis; secretion of IL-6 and IL-8 by astrocytes	Melatonin MT1/MT2 receptor agonist, NLRP3 inhibitor, JNK inhibitor, oxytocin, Baicalin, Xanthohumol, ketamine, Serp-1	[18,19,20,21,22,23,24,25]
IL-3	Inhibition of secondary degeneration	Interleukin-3 (IL-3) and granulocyte/macrophage colony-stimulating factor (GM-CSF)	[26]
IL-4	Matter integrity promotion; long-term neurological recovery	Melatonin MT1/MT2 receptor agonist, Mesenchymal stem/stromal cells therapy	[20,22]
IL-6	Nerve growth factor production	NLRP3 inhibitor, TGF-β1 infusion, metformin, melatonin, Vitamin D, JNK inhibitor, exosomes, lipopolysaccharide (LPS) injection, mesenchymal stem/stromal cells therapy	[18,19,20,21,22,23,27,28,29]
IL-7	Induction of gliosis	Lipopolysaccharide (LPS) injection	[30]
IL-9	excitotoxic damage; destruction of BBB		[31]
IL-10	Downregulation of pro-inflammatory cytokines	Melatonin MT1/MT2 receptor agonist, lipopolysaccharide (LPS) injection, mesenchymal stem/stromal cells therapy, statins, formononetin, Serp-1	[19,20,22,26]
IL-16	Lymphocytes and microglia activation; accumulation in cerebral vessels	anti-IL-16 antibody	[32]
IL-17	Neutrophils encroachment	Monoclonal antibodies	[33]
IL-18	Caspase-1 activation	Exosomes, NLRP3 inhibitor	[18,29]
IL-23	Leads to neurologic deficits	Monoclonal antibodies	[33]
CHEMOKINES		CXCL immunotherapy, glucagon-like peptide-1 receptor (GLP-1R) agonist	[34,35]
CXCL1	Neutrophil circulation into the brain		
CXCL3	Migration of neutrophils across epithelial barriers		
CXCL5	Microglia activation; BBB damage; astrogliosis		
CXCL8	Neutrophil infiltration into brain parenchyma		
CXCL9	Inhibition of selected T cells		
CXCL10	Blood-derived monocytes promotion (to accumulate around perivascular vessels)		
